# Integration of a blood return strategy into the double plasma molecular adsorption system

**DOI:** 10.1111/sdi.13119

**Published:** 2022-07-12

**Authors:** Maoliang Fu, Xinyan Liu, Wenwen Meng, Ranran Dong, Xihua Geng, Xuan Song

**Affiliations:** ^1^ Department of Critical Care Medicine Dong E Hospital Liaocheng China; ^2^ Department of Critical Care Medicine Shandong Provincial Hospital Affiliated to Shandong First Medical University Jinan China

Dear Editor,

The double plasma molecular adsorption system (DPMAS) is a kind of a biotic artificial liver.^1^ Its clinical application value is constantly expanding, particularly in cases of plasma deficiencies. Many commonly used blood purification devices (e.g., Fresenius Multifiltrate and Baxter Prismaflex) do not currently have built‐in DPMAS treatment modes and require pipeline modification. Therefore, there is no unified standardized blood return procedure at the end of treatment. Current methods of blood return and their disadvantages include the following: (1) air return method: this method could increase the risk of infection^2^; (2) plasma circulation pipeline discard method: as this method will lead to the loss of plasma, exogenous plasma needs to be supplemented that could increase medical expenses and blood product consumption; (3) substitution fluid pump return method: operation of the substitution fluid pump is relatively complex, and many of the pipeline disassembly and assembly steps require strict aseptic practices. In order to standardize the blood return operation procedure in treatment using DPMAS, we introduce a new DPMAS pipeline installation method utilizing an integrated blood return strategy, which has been successfully deployed in clinical practice. The details are as follows:

## IMPROVED DPMAS PIPELINE INSTALLATION METHOD

Original pipeline installation method: the patient's blood passes through a plasma separator to isolate the plasma, and the blood cell components enter the blood return pipeline. The separated plasma first passes through the disposable plasma bilirubin adsorption column (BS330), then through the disposable hemoperfusion cartridges (HA330‐II), and is then recombined with the blood cells and returned to the patient.

**FIGURE 1:**
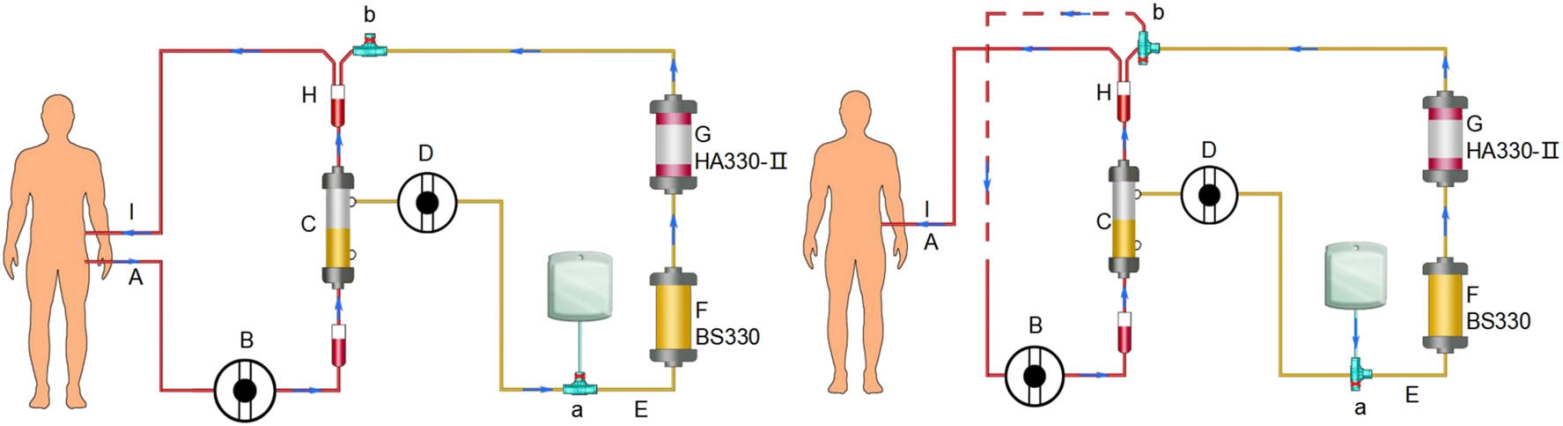
Diagram of improved DPMAS pipeline installation and integrated blood return strategy. (A) Blood outflow, (B) blood pump, (C) plasma separator, (D) plasma pump, (E) disposable blood loop catheter, (F) BS330, (G) HA330‐II, (H) re‐mixing container, (I) blood return. (a) Three‐way valve 1, (b) three‐way valve 2. For blood return, turn the second three‐way valve before the re‐mixing container, to connect the blood outflow tubing to one side branch of the three‐way valve. The first three‐way valve is rotated to allow flow of 0.9% normal saline into the disposable blood loop catheter. This allows blood to flow from the 0.9% normal saline to the blood return side.

In order to integrate blood return, we added two three‐way valves in the pipeline. The first is located between the plasma pump and the BS330 column. The upper portion of this three‐way valve is connected to a bag containing 500 mL 0.9% normal saline using a disposable infusion connector set. During the treatment, 0.9% normal saline access and this three‐way valve remains closed. The second valve is located further along the disposable blood loop catheter between the HA330‐II and the re‐mixing container. The unconnected side of the three‐way valve is closed to ensure smooth flow between the plasma separation system and the plasma adsorption system (Figure 1).

## INTEGRATED BLOOD RETURN STRATEGY

After DPMAS treatment, the plasma separation system is stopped first and then turning off the blood pump. Under aseptic operation, turn the second three‐way valve before the re‐mixing container, to connect the blood outflow tubing to one side branch of the three‐way valve. The first three‐way valve is rotated to allow flow of 0.9% normal saline into the disposable blood loop catheter. This allows blood to flow from the 0.9% normal saline to the blood return side (Figure 1). Following this conversion of the original parallel flow system to a serial flow system, the blood pump is restarted, and the blood is returned at the speed of 100 mL/min. Once all of the blood has been returned, the treatment is finished.

## KEY POINTS OF INTEGRATED BLOOD RETURN STRATEGY

(1) Strict attention should be paid to the opening direction of the three‐way valves to ensure that the serial blood return pipeline remains open. (2) The blood return procedure should be adjusted according to the different device models. For example, the Fresenius Multifiltrate system can first turn off the plasma pump and return blood in the treatment mode. It can also adjust the blood return setting before treatment and directly enter “End of Treatment” mode for blood return. The Baxter Prismaflex system allows direct blood return in blood return mode. (3) Any blood return procedures should be carried out under strict aseptic conditions.

With its rapid clearance of bilirubin, inflammatory mediators, and other accumulated substances, DPMAS has been widely used in many cases of liver failure, hyperbilirubinemia due to hemolytic anemia, hepatic encephalopathy, perioperative treatment of liver transplantation, multiple organ dysfunction syndrome, and sepsis.^3^, ^4^ Studies report that in the treatment of severe COVID‐19, it has been successfully applied for the adsorption and clearance of inflammatory factors and achieved some efficacy.^5^ Especially in the case of blood deficiency, DPMAS becomes the preferred treatment mode.

The required modifications consist only of the installation of two three‐way valves in the original pipeline and requires no high‐priced consumables. Clinical application of this integrated blood return strategy could solve the problem of difficult blood return, reduce pipeline split times, decrease infection risk caused by blood leakage, and reduce complications caused by blood loss and blood transfusion. As a result, patient satisfaction and medical staff efficiency can be improved. This strategy is simple to apply and can be widely used in clinical practice.
